# Department of Canine, Feline, and Avian Medicine and Surgery

**Published:** 1902-06

**Authors:** Cecil French

**Affiliations:** D.V.S. (McGill and Munich), Washington, D. C.


### DEPARTMENT OF CANINE, FELINE, AND AVIAN MEDICINE AND SURGERY.

By Cecil French, D.V.S. (McGill and Munich),
WASHINGTON, D. C.

Relative Susceptibility to Follicular Mange. Some recent observations on follicular mange have led me to believe that the young of the domestic dog possesses a greater predisposition to infestation by the parasite acarus folliculorum than does the adults. A Great Dane bitch, aged eighteen months, in perfect health, without any sign of skin trouble, and which with its mate had been in isolation from other dogs for over a year, gave birth to a large litter. Ten weeks later one of the pups was shipped to a distance of some five hundred miles, and another was obtained by myself and introduced to my own kennel, being placed in the company of another Dane bitch, aged four years, with a puppy of its own, aged eight weeks.

At that time every animal was apparently in perfect health. Three weeks later I observed the hair of the head of the new-comer to be falling out. Closer examination showed the head and front of the neck to be diffusely inflamed with a few minute, isolated pustules. Microscopical examination of the contents of the pustules revealed the presence of the acarus folliculorum.

Understanding the almost ineradicable nature of this disease when diffuse, I immediately removed this animal and destroyed it. At the same time I inspected the remainder of the litter and found everyone of the puppies similarly affected. The mother of the litter, though still running with her offspring, showed no trace of the disease.

I next closely examined the puppy of the second bitch, but found the skin clean and free of any disturbance. This animal and its mother were removed to new quarters, in order that they might be far from any possible source of infestation in the old. Four weeks later this puppy also developed the disease, and about the same time I received word that the puppy which had been sent away at the beginning was also infested.

Neither adult bitch has shown any trace of the disease up to the present, a period of two months since the second puppy was destroyed.

These cases would seem to demonstrate that puppies are highly susceptible to infestation by the follicular parasite; that the disease is very contagious among them; that adults may enjoy freedom from infestation amounting almost to immunity, and that the parasite itself may exist in the soil in localities where it is indigenous.

Remedy for Circumscribed Follicular Mange. A case presenting two isolated patches of this disease on the head was treated with Peruvian balsam and Tuson's tincture of iodine, these two medicaments being used on alternate days. At the end of four weeks' constant application the diseased areas took on a normal appearance, and the hair commenced to grow. There has been no return of the trouble, and two years have elapsed since the outbreak.

Goitre Cured by Iodine Medication. A greyhound bitch, aged eleven years, was brought to me suffering from a large follicular goitre of the left side. Breathing was much impeded, and the animal was considerably distressed at times. I prescribed 5-grain pills of potassium iodide, to be administered every two hours. This treatment was kept up continuously until the animal had received 400 pills, or a total of over 4 ounces of the drug, when she was entirely cured of the enlargement. Beyond a slight reddening and weeping of the conjunctiva no unpleasant effect was produced.

Unusual Exhibitions of the Maternal Instinct. I have already reported in the columns of this Journal instances of the suckling of puppies by a cat and of kittens by a dog, but the following cases are decidedly more remarkable:

A man by the name of Price, living at Dickinson, near Boyd, Md , owns a cat that has adopted five young rabbits, and is nursing them along with three of her own kittens.

In the April number of the *Cat Journal* there is an account of the adoption of a family of young rats by a cat, and accompanying the same is the incontrovertible evidence of a photograph.

Mr. S. C. Cross, of Great Cacapon, W. Va., has in his possession a female cat which is at the present time suckling four young gray foxes.

Miss Burrint, proprietor of the Columbia Cattery, at Washington City, reports owning a cat which is nursing its own litter together with a young guinea-pig.

